# Supplementary figures and images for: No sex difference in preen oil chemical composition during incubation in Kentish plovers
Source: PeerJ. 2024 May 8;12:e17243. doi: 10.7717/peerj.17243 (PMC11088368; doi:10.7717/peerj.17243)

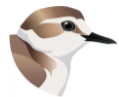

Female

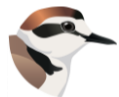

Male

# Samples

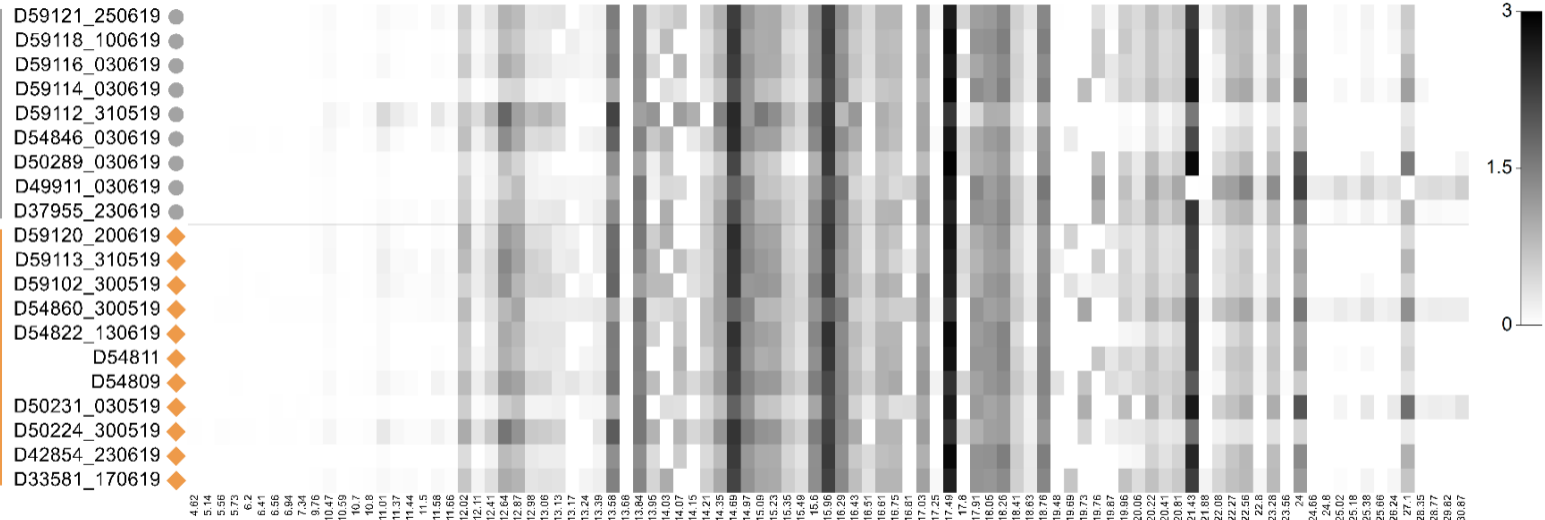

Chemical substances (retention time)

Supplement: Supplemental Information 1 — Chemical substances are identified by their retention time (min). [file peerj-12-17243-s001.pdf]

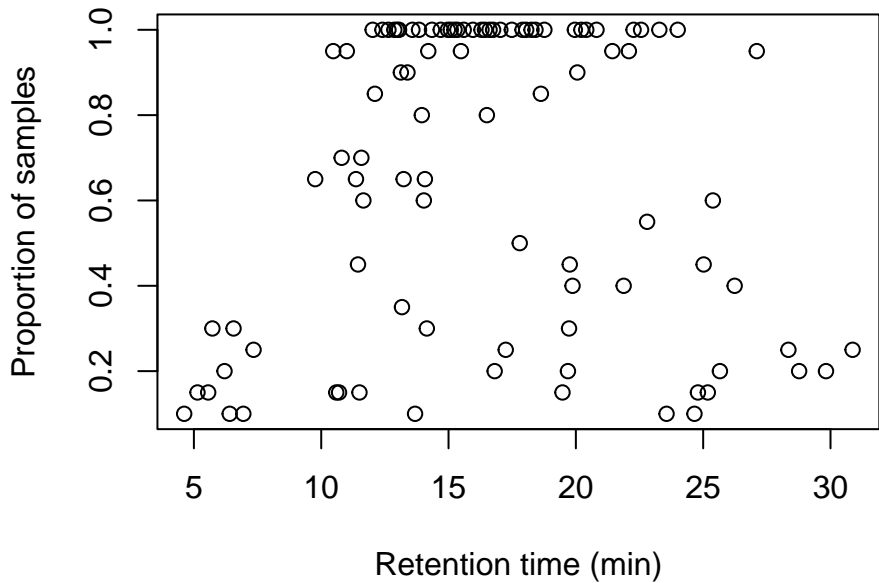

Supplement: Supplemental Information 2 — Each circle represents a putative chemical substance. [file peerj-12-17243-s002.pdf]
