# Supplementary material for: No sex difference in preen oil chemical composition during incubation in Kentish plovers
Source: PeerJ. 2024 May 8;12:e17243. doi: 10.7717/peerj.17243 (PMC11088368; doi:10.7717/peerj.17243)
Supplement: Supplemental Information 3 — Chemical substances were defined by their retention time and are presented in descending order of mean relative abundance. Substances were first putatively identified at the substance level by comparing mass spectrometry (MS) with the NIST library, and then the class of the putatively identified substances was identified if it was matching across all samples. Our analytical method did not allow for an accurate identification of substance names (therefore not reported here). [file peerj-12-17243-s003.docx]

**Table S1.** Chemical substances detected in the preen oil samples of female and male adult Kentish plovers during incubation (*N* = 20 samples). Chemical substances were defined by their retention time and are presented in descending order of mean relative abundance. Substances were first putatively identified at the substance level by comparing mass spectrometry (MS) with the NIST library, and then the class of the putatively identified substances was identified if it was matching across all samples. Our analytical method did not allow for an accurate identification of substance names (therefore not reported here).

| **Substance** (retention time in min) | ***N* samples** containing the substance | **Relative abundance**  (mean ± SD) | **Putative class** |
| --- | --- | --- | --- |
| 17.49 | 20 | 13.70 ± 2.58 | Monoester |
| 21.43 | 19 | 8.86 ± 3.87 | Monoester |
| 15.96 | 20 | 8.53 ± 1.15 | Monoester |
| 14.69 | 20 | 8.50 ± 1.87 | Monoester |
| 13.58 | 20 | 3.69 ± 1.59 | Monoester |
| 13.84 | 20 | 3.16 ± 0.69 | Monoester |
| 18.76 | 20 | 2.93 ± 0.53 | Unidentified |
| 24.00 | 20 | 2.81 ± 2.00 | Monoester |
| 16.29 | 20 | 2.80 ± 0.59 | Unidentified |
| 18.26 | 20 | 2.72 ± 0.52 | Unidentified |
| 15.60 | 20 | 2.70 ± 0.57 | Monoester |
| 14.97 | 20 | 2.57 ± 0.47 | Unidentified |
| 18.05 | 20 | 2.23 ± 0.28 | Unidentified |
| 17.91 | 20 | 2.07 ± 0.58 | Monoester |
| 12.64 | 20 | 1.91 ± 1.14 | Monoester |
| 17.03 | 20 | 1.85 ± 0.33 | Monoester |
| 15.09 | 20 | 1.83 ± 0.66 | Monoester |
| 15.24 | 20 | 1.70 ± 0.52 | Monoester |
| 14.35 | 20 | 1.69 ± 0.56 | Unidentified |
| 12.87 | 20 | 1.40 ± 0.49 | Alkene |
| 16.61 | 20 | 1.26 ± 0.23 | Monoester |
| 27.11 | 19 | 1.22 ± 1.16 | Monoester |
| 22.56 | 20 | 1.20 ± 0.61 | Unidentified |
| 16.75 | 20 | 1.12 ± 0.34 | Monoester |
| 16.43 | 20 | 1.11 ± 0.40 | Monoester |
| 23.28 | 20 | 1.11 ± 0.53 | Ether |
| 20.22 | 20 | 0.99 ± 0.38 | Unidentified |
| 22.27 | 20 | 0.92 ± 0.43 | Unidentified |
| 12.02 | 20 | 0.89 ± 0.35 | Unidentified |
| 20.81 | 20 | 0.80 ± 0.37 | Monoester |
| 15.35 | 20 | 0.74 ± 0.24 | Monoester |
| 18.42 | 20 | 0.67 ± 0.15 | Monoester |
| 13.95 | 16 | 0.66 ± 0.62 | Monoester |
| 14.07 | 13 | 0.66 ± 0.63 | Unidentified |
| 14.21 | 19 | 0.66 ± 0.25 | Unidentified |
| 14.03 | 12 | 0.61 ± 0.62 | Unidentified |
| 19.96 | 20 | 0.60 ± 0.35 | Monoester |
| 20.41 | 20 | 0.52 ± 0.22 | Unidentified |
| 15.49 | 19 | 0.46 ± 0.16 | Unidentified |
| 12.41 | 20 | 0.45 ± 0.3 | Monoester |
| 19.76 | 9 | 0.45 ± 0.64 | Unidentified |
| 22.08 | 19 | 0.45 ± 0.40 | Unidentified |
| 12.98 | 20 | 0.43 ± 0.27 | Monoester |
| 13.06 | 20 | 0.43 ± 0.31 | Monoester |
| 16.51 | 16 | 0.36 ± 0.21 | Monoester |
| 20.06 | 18 | 0.34 ± 0.19 | Monoester |
| 19.73 | 6 | 0.32 ± 0.59 | Unidentified |
| 13.13 | 18 | 0.31 ± 0.22 | Monoester |
| 17.80 | 10 | 0.25 ± 0.28 | Monoester |
| 18.63 | 17 | 0.21 ± 0.11 | Unidentified |
| 14.15 | 6 | 0.19 ± 0.38 | Unidentified |
| 13.39 | 18 | 0.17 ± 0.10 | Monoester |
| 12.11 | 17 | 0.14 ± 0.13 | Monoester |
| 25.38 | 12 | 0.13 ± 0.18 | Unidentified |
| 11.01 | 19 | 0.12 ± 0.13 | Monoester |
| 16.81 | 4 | 0.11 ± 0.24 | Unidentified |
| 19.69 | 4 | 0.11 ± 0.27 | Monoester |
| 19.87 | 8 | 0.11 ± 0.15 | Unidentified |
| 13.24 | 13 | 0.09 ± 0.08 | Monoester |
| 22.80 | 11 | 0.09 ± 0.09 | Monoester |
| 26.24 | 8 | 0.08 ± 0.12 | Unidentified |
| 30.87 | 5 | 0.08 ± 0.19 | Monoester |
| 11.58 | 14 | 0.07 ± 0.08 | Unidentified |
| 13.17 | 7 | 0.07 ± 0.11 | Monoester |
| 25.02 | 9 | 0.07 ± 0.13 | Monoester |
| 10.47 | 19 | 0.05 ± 0.03 | Unidentified |
| 28.35 | 5 | 0.05 ± 0.11 | Unidentified |
| 28.77 | 4 | 0.05 ± 0.14 | Unidentified |
| 29.82 | 4 | 0.05 ± 0.12 | Unidentified |
| 11.37 | 13 | 0.04 ± 0.06 | Monoester |
| 17.25 | 5 | 0.03 ± 0.05 | Unidentified |
| 19.48 | 3 | 0.03 ± 0.09 | Unidentified |
| 21.88 | 8 | 0.03 ± 0.06 | Unidentified |
| 25.66 | 4 | 0.03 ± 0.09 | Monoester |
| 11.44 | 9 | 0.02 ± 0.03 | Unidentified |
| 11.66 | 12 | 0.02 ± 0.03 | Unidentified |
| 24.66 | 2 | 0.02 ± 0.06 | Unidentified |
| 24.80 | 3 | 0.02 ± 0.07 | Monoester |
| 25.18 | 3 | 0.02 ± 0.07 | Monoester |
| 9.76 | 13 | 0.01 ± 0.01 | Unidentified |
| 10.80 | 14 | 0.01 ± 0.02 | Unidentified |
| 11.50 | 3 | 0.01 ± 0.01 | Unidentified |
| 13.69 | 2 | 0.01 ± 0.03 | Monoester |
| 23.56 | 2 | 0.01 ± 0.02 | Unidentified |
| 4.62 | 2 | < 0.01 | Unidentified |
| 5.14 | 3 | < 0.01 | Unidentified |
| 5.56 | 3 | < 0.01 | Unidentified |
| 5.73 | 6 | < 0.01 | Fatty acid |
| 6.20 | 4 | < 0.01 | Unidentified |
| 6.41 | 2 | < 0.01 | Unidentified |
| 6.56 | 6 | < 0.01 | Unidentified |
| 6.94 | 2 | < 0.01 | Fatty acid |
| 7.34 | 5 | < 0.01 | Unidentified |
| 10.59 | 3 | < 0.01 | Monoester |
| 10.70 | 3 | < 0.01 | Unidentified |
