# Supplementary material for: No sex difference in preen oil chemical composition during incubation in Kentish plovers
Source: PeerJ. 2024 May 8;12:e17243. doi: 10.7717/peerj.17243 (PMC11088368; doi:10.7717/peerj.17243)
Supplement: Supplemental Information 4 — We tested the effect of sex, the number of days after laying and the interaction between sex and the number of days after laying on Bray-Curtis dissimilarities in preen oil composition in Kentish plovers. The PERMANOVA was run on 16 samples (N = 16; 7 females and 9 males) out of the 20 samples, excluding 4 samples (2 females and 2 males) among “paired” samples (i.e. from the same breeding pair), so that all samples were from different breeding pairs. The PERMANOVA was iterated 1,000 times with a randomized selection of four excluded samples at each iteration, and was run with 9,999 permutations and sequential (type I) sums of square. Sums of square (SS), R2 and F values are reported as median (interquartile range) across iterations. [file peerj-12-17243-s004.docx]

**Table S2.** Results from permutational multivariate analysis of variance (PERMANOVA) testing the effect of sex, the number of days after laying and the interaction between sex and the number of days after laying on the beta diversity (Bray-Curtis dissimilarities) of the preen oil of Kentish plovers. The PERMANOVA was run on 16 samples (*N* = 16; 7 females and 9 males) out of the 20 samples, excluding 4 samples (2 females and 2 males) among “paired” samples (i.e. from the same breeding pair), so that all samples were from different breeding pairs. The PERMANOVA was iterated 1,000 times with a randomized selection of four excluded samples at each iteration, and was run with 9,999 permutations and sequential (type I) sums of square. Sums of square (*SS*), *R^2^* and *F* values are reported as median (interquartile range) across iterations.

| **Bray-Curtis dissimilarity** | df | SS | *R^2^* | *F* | *P* |
| --- | --- | --- | --- | --- | --- |
| Sex | 1 | 0.02 (0.01–0.03) | 0.11 (0.03–0.13) | 1.77 (0.57–2.45) | 0.35 |
| Days after laying | 1 | 0.02 (0.00–0.03) | 0.11 (0.01–0.15) | 1.79 (0.27–2.87) | 0.48 |
| Sex × Days after laying | 1 | 0.01 (0.01–0.04) | 0.06 (0.02–0.14) | 1.06 (0.86–2.05) | 0.34 |
| Residuals | 12 | 0.16 (0.14–0.20) | 0.73 (0.64–0.80) | **—** | **—** |
