# Supplementary material for: No sex difference in preen oil chemical composition during incubation in Kentish plovers
Source: PeerJ. 2024 May 8;12:e17243. doi: 10.7717/peerj.17243 (PMC11088368; doi:10.7717/peerj.17243)
Supplement: Supplemental Information 5 — We tested the effect of sex (fixed), the number of days after laying (fixed), the interaction between sex and number of days after laying (fixed) and pair ID (random) on the chemical diversity (Shannon index) and richness (number of substances) of the preen oil of Kentish plovers (N = 20 samples; 9 females and 11 males). [file peerj-12-17243-s005.docx]

**Table S3.** Results from linear mixed models (LMMs) testing the effect of sex (fixed), the number of days after laying (fixed), the interaction between sex and number of days after laying (fixed) and pair ID (random) on the alpha diversity, namely chemical diversity (Shannon index) and richness (number of substances), of the preen oil of Kentish plovers (*N* = 20 samples; 9 females and 11 males).

| **Chemical diversity** | β [95% CI] | Variance |
| --- | --- | --- |
| Sex (male) | 0.09 [–0.15, 0.36] | **—** |
| Days after laying | 0.00 [–0.01, 0.01] | **—** |
| Sex × Days after laying | 0.00 [–0.02, 0.01] | **—** |
| Pair ID | **—** | 0 |
|  |  |  |
| **Chemical richness** | β [95% CI] | Variance |
| Sex (male) | 5.88 [–12.7, 26.4] | **—** |
| Days after laying | 0.02 [–0.81,0.86] | **—** |
| Sex × Days after laying | –0.32 [–1.73, 0.93] | **—** |
| Pair ID | **—** | 0 |
